# Polype fibro-épithélial géant de l'anus sur grossesse: à propos d'un cas

**DOI:** 10.11604/pamj.2019.33.300.19815

**Published:** 2019-08-16

**Authors:** Seydou Sogoba, Mamadou Ibrahima Kampo, Bourama Coulibaly, Tiounkani Théra, Djibril Kassogué

**Affiliations:** 1 Hôpital de Tombouctou, Tombouctou, Mali; 2 Service d’Anatomie et Cytologie Pathologiques du CHU du Point G, Bamako, Mali; 3 Université des Sciences, des Techniques et des Technologies de Bamako (USTTB); 4 Service de Gynécologie Obstétrique du CHU du Point G, Bamako, Mali

**Keywords:** Tumeur anale, polype fibro-épithélial, grossesse, Anal tumor, fibroepithelial polyp, pregnancy

## Abstract

Le polype fibro-épithélial anal est une tumeur bénigne rarement rapportée au cours de la grossesse. Nous rapportons un cas de polype fibro épithélial géant de l'anus chez une multipare de 31 ans. L´apparition a été progressive depuis plus de 3 ans. L'examen découvrait une formation bourgeonnante, de consistance ferme, à base sessile, appendue à la marge anale sur une grossesse normalement évolutive de 31 semaines d'aménorrhée. L'examen endo-anal était sans particularité. Une exérèse chirurgicale a été réalisée sous rachianesthésie. La pièce opératoire est une formation unique de 21x12x7cm recouverte par la peau. L'examen histologique a objectivé un polype fibro-épithélial anal sans signe de malignité. Les suites opératoires ont été simples.

## Abstract (en)

Anal fibroepithelial polyp is a benign tumor rarely reported during pregnancy. We here report a case of giant anal fibroepithelial polyp in a 31-year old multiparous woman. It developed progressively over the previous 3 years. Clinical examination showed firm sessile bud formation, at the anal margin. The patient had normal ongoing pregnancy (31 weeks of amenorrhea). Endoanal examination was normal. Surgical resection was preformed under spinal anesthesia. The specimen measured 21x12x7cm and was covered by the skin. Histological examination showed anal fibroepithelial polyp without signs of malignancy. The postoperative course was uneventful.

## Introduction

Le polype fibreux épithélial anal est une lésion rare mais bien décrite dans la littérature au cours de la grossesse [1]. Il s'agit d'une tumeur bénigne de l'anus, donc une masse qui ne se ne propage pas à d'autres parties du corps (pas de métastases). Les tumeurs non cancéreuses ne mettent habituellement pas la vie en danger. L'examen est clinique et le diagnostic est histologique après une exérèse chirurgicale [2].

## Patient et observation

**Figure 1:**
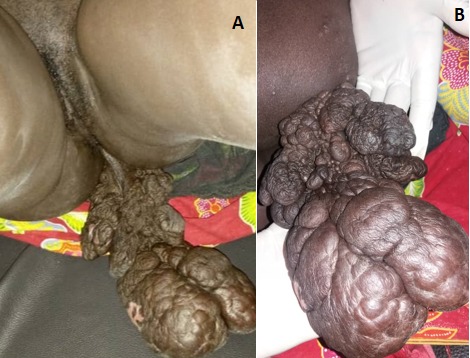
Aspect macroscopique de la tumeur anale fibro-épithéliale géante: A) vue de face, patiente en position gynécologique et B) vue de dos, patiente en décubitus latéral)

**Figure 2:**
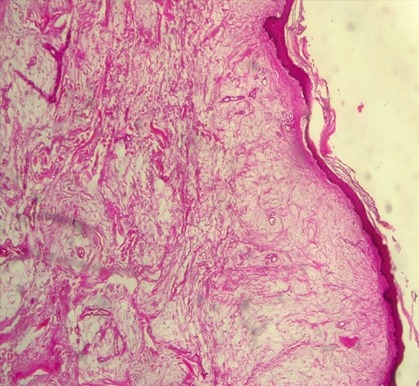
HE x 100: aspect microscopique d’un polype fibro-épithélial montrant une formation polypoïde tapissée par une muqueuse malpighienne régulière

**Figure 3:**
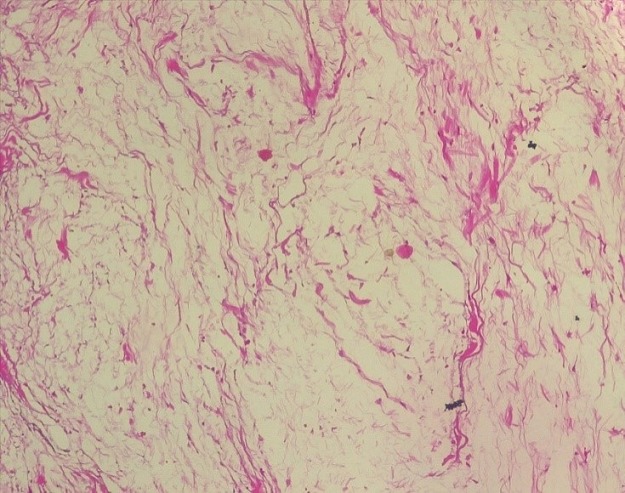
HE x 100: aspect microscopique d’un polype fibro-épithélial montrant un tissu conjonctif œdémateux, myxoïde et richement vascularisé sans atypie cytonucléaire

Patiente de 31 ans G5 P4 ménagère, venue d'elle même à la maternité de l'hôpital de Tombouctou pour une grosse masse entre les cuisses sur grossesse de terme imprécis. Il s'agit d'une grossesse non suivie, aucun bilan biologique ni échographique n'a été réalisé. La masse serait apparue il y a plus de trois ans, avec augmentation progressive de volume, d'évolution plus rapide pendant la grossesse. L'examen à l'admission note un bon état général, des constantes hémodynamiques stables. L'examen obstétrical est marqué par une hauteur utérine à 28 cm, présentation céphalique, des bruits du cœur fœtal à 140 battements par minute au Sonicaïd. Au niveau de la vulve, une grosse masse d'aspect fibromateux pédiculée est développée sur le périnée postérieur proche de l'anus (Figure 1). L'examen des autres appareils ne note pas de particularité. L'échographie réalisée en urgence objective une grossesse mono fœtale intra utérine évolutive de 31 semaines d'aménorrhée + 3 jours. Le bilan sanguin pré opératoire note un groupe sanguin O rhésus positif, les sérologies HIV, BW (Bordet Wassermann) étaient négatives, le taux hémoglobine: 12,9g/l, le bilan d'hémostase faisable (TS-TC) était normal. La glycémie en jeun à 108mg/dl a révélé un diabète gestationnel. Une résection de la tumeur a été réalisée au bistouri à froid et électrique après clampage, hémostase satisfaisante, suture faite au Vicryl puis des points séparés avec le fil à peau, pansement propre. La pièce opératoire a été adressée au servie d'anatomie pathologique. Les suites opératoires ont été simples avec sortie de la patiente au quatrième jour d'hospitalisation. A l'examen macroscopique la pièce de tumorectomie mesurait 21x12x7cm recouverte par la peau, de consistance ferme, blanc-jaunâtre. Sur le plan histologique il s'agissait d'une formation polypoide tapissée par une muqueuse malpighienne sans particularité. L'épithélium reposait sur un tissu conjonctif richement vascularisé. Ce tissu conjonctif est œdémateux et myxoide. Absence d'atypie cytonucléaire (Figure 2 et Figure 3).

## Discussion

**Fréquence**: le polype fibro épithélial géant de l'anus sur grossesse est une lésion rare dont la fréquence exacte n'est pas connue car très peu rapportée dans la littérature.

**Diagnostic et prise en charge**: dans notre cas le polype fibro épithélial géant de l'anus a été pris en charge tardivement sur la grossesse. Plusieurs insuffisances concourent au retard dans la prise en charge: l'absence de suivi médical et de consultations prénatales, les difficultés à accéder aux centres de santé à cause de l'insécurité, la faible expertise du personnel en diagnostic et dépistage des pathologies tumorales. La réduction de ce gap important dans le retard de prise en charge passera par une meilleure fréquentation des centres de santé par les malades, la formation du personnel soignant et une plus grande implication des autorités pour assurer la sécurité des personnes dans les zones de conflit.

Le bilan minimum réalisable à notre niveau nous a permis d'effectuer une exérèse tumorale en un premier temps et dans un deuxième temps la pièce opératoire a été adressée à une autre structure pour l'examen histologique. Le bilan hormonal et immunologique a été envisagé mais était non réalisable à cause du faible plateau technique dans notre contexte. En effet, la présence de récepteurs hormonaux a été rapportée [3, 4] et favoriserait la croissance de ces polypes au cours de la grossesse. Le traitement des polypes fibro-épithéliaux de l'anus (PFEA) sur grossesse est l'exérèse mais son caractère incomplet, ainsi que l'hormonodépendance, expliquerait les récidives. Selon certains auteurs ces formations ne représentent probablement pas de véritables tumeurs, mais plutôt une manifestation d'hyperplasie du tissu conjonctif sous épithélial à l'induction hormonale, particulièrement dans les localisations vulvovaginales d'autres auteurs y voient un stade final d'organisation d'un bourgeon charnu [3, 5]. La prise en charge chirurgicale du polype fibro épithélial géant de l'anus par polypectomie n'a pas eu d'influence sur l'évolution normale de la grossesse dans notre cas.

## Conclusion

le polype fibro-épithélial géant de l'anus sur grossesse est une lésion bénigne rare. Son traitement efficace est possible par une exérèse complète même avec des ressources limitées. Le pronostic est bon et la guérison est obtenue après une exérèse complète.

## Conflits d’intérêts

Les auteurs ne déclarent aucun conflit d'intérêts.
